# A Case Study for the Extraction, Purification, and Co-Pigmentation of Anthocyanins from *Aronia melanocarpa* Juice Pomace

**DOI:** 10.3390/foods11233875

**Published:** 2022-12-01

**Authors:** Maria Cinta Roda-Serrat, Behnaz Razi Parjikolaei, Mehrdad Mohammadifakhr, Juncal Martin, Birgir Norddahl, Massimiliano Errico

**Affiliations:** 1 Department of Green Technology, University of Southern Denmark, Campusvej 55, DK-5230 Odense, Denmark; 2 Membrane Science and Technology Cluster, Department of Science and Technology, Mesa+ Institute for Nanotechnology, University of Twente, P.O. Box 217, 7500 AE Enschede, The Netherlands

**Keywords:** *Aronia melanocarpa*, anthocyanin, enzyme-assisted extraction, co-pigmentation

## Abstract

Chokeberry (*Aronia melanocarpa*) pomace is a by-product from the juice industry very rich in anthocyanins and other bioactive components. Recovery and purification of anthocyanins from the pomace is a viable valorization strategy that can be implemented to produce high-value natural food colorants with antioxidant properties. In this study, chokeberry pomace was subjected to enzyme-assisted extraction using commercial pectinases. The extracts were further purified by adsorption–desorption using an acrylic resin and stabilized by co-pigmentation with ferulic acid. The anthocyanin concentration and antioxidant activity of the extracts were unaffected by the enzymatic treatment at the conditions tested. The total phenolic content of the extracts suffered minor variations depending on the enzyme formulation used, whereas the dissolved solid content increased in all cases. The adsorption–desorption strategy allowed a 96% recovery of the anthocyanins initially present in the extract, whereas the co-pigmentation treatment magnified the intensity of the color in terms of absorbance, and improved the stability during storage up to one month.

## 1. Introduction

In juice production, a large part of the fruit weight remains as pomace after the pressing step. This pomace consists mainly of the insoluble parts of the fruit, namely residual fruit flesh, skin, and seeds. Similar to other organic crop residues, fruit pomace is sometimes used as animal feed, composted, or used for biogas production [1]. However, the pomace generally contains large quantities of macronutrients and phytochemicals that can be further recovered and incorporated into new products [2,3].

Of particular interest are anthocyanins, a family of water-soluble pigments that give their red, violet, and blue color to many flowers, fruits, and vegetables. Due to their coloring abilities, anthocyanins find application as natural food colorants [4], substituting artificial coloring agents that are suspected to have adverse effects in children [5,6]. Consumption of dietary anthocyanin may be beneficial to human health due to their various bioactive properties such as antioxidant, anticarcinogenic, cardioprotective, and antidiabetic activities [7,8,9]. Furthermore, their antioxidant capacity makes anthocyanin-based natural colorants suitable as food antioxidants able to extend the shelf life and increase the nutritional value of processed foods [10].

The classical method for recovering anthocyanins from plant matrices is solvent extraction using acidified aqueous methanol, ethanol, or acetone [11]. Using pure water at room temperature results in lower extraction levels than the organic solvent–aqueous mixtures in the same conditions [12]. However, the extraction yield can be improved by techniques such as ultrasound treatment [13], pressurized-liquid extraction [14], pulsed electric field treatment [15], or enzymatic degradation of the plant cell wall [16].

Enzyme-assisted extraction of plant bioactive components offers several advantages such as mild conditions, no need for flammable, volatile, or hazardous solvents, and high substrate specificity [16]. There is no doubt that the recovery of anthocyanins from plant sources and biowaste has attracted a lot of attention in the recent years [17,18,19]. This tendency results from the increasing awareness for more natural foods and healthy ingredients, which has transformed the global food and beverage industry. The main challenge in the industrial production and application of anthocyanins as natural colorants is their relatively low stability. In order to minimize degradation, aqueous solutions of anthocyanins need to be stored at low pH and low temperature [20]. A stabilization technique that has received attention throughout the years is co-pigmentation, in which pigments interact non-covalently with colorless components forming complexes that shield them from nucleophilic attack and subsequent degradation [21,22]. This complexation often results in color enhancement by increasing the absorbance intensity (hyperchromic shift), or in alteration of the color hue by shifting the absorption maximum wavelength (bathochromic shift) [23]. These spectral modifications are attributed to intermolecular interactions such as π-π overlapping between aromatic residues, dipole-dipole interactions, and hydrogen bonding [21].

Black chokeberry (*Aronia melanocarpa*) stands out as one of the berries with highest anthocyanin content [9,10]. In their review, Denev et al. [7] reported that the total anthocyanin content in chokeberries from different sources was between 428 and 1790 mg/100 g fresh weight (FW). Years later, Denev et al. [24] measured the composition of chokeberries from different growers in Bulgaria and reported large variations for most components, even though all berries where of the same variety, and had been harvested in the same period of the year. In the case of total anthocyanin content, it was in the range 284–686 mg/100 g FW. That study focused on the production of chokeberry juices and nectars, and observed that processing at high temperature increased the anthocyanin and total polyphenol content. However, it resulted in higher astringency in the taste of the juice, caused by the coextraction of tannins. Because of this phenomenon, chokeberry juice is in some cases produced at relatively low temperatures, and the juice processing waste, or pomace, remains a rich source of anthocyanins. The chokeberry pomace can thus have very different anthocyanin content depending on the juice production methodology used [25], and has been reported to be in the range 5–20 mg/g dry basis [19,26,27], with some cases in the higher range of 66–115 mg/g dry basis [28,29].

Chokeberry pomace could in principle be dried and ground to be used directly as nutritional powder rich in dietary fiber and antioxidants [30]. However, one or more high value products can be formulated via extraction and purification of the valuable components. Most of the studies focusing on anthocyanin recovery from chokeberry pomace utilize ultrasound-assisted extraction in methanol– or ethanol–water mixtures [27,29]. A study by Roda-Serrat et al. [31] presented a method for anthocyanin recovery by homogenization in citric acid aqueous solutions that could achieve up to 70% of the total anthocyanin extractable by methanol. Vauchel et al. [32] demonstrated the possibility of integrating the extraction and adsorption processes by a combined hybrid process in pilot scale, highlighting the versatility and also scalability of this type of processes.

In the present work, enzyme-assisted extraction of chokeberry pomace was performed in aqueous buffers. Four commercially available enzyme formulations were tested; their influence on anthocyanin concentration, total phenolic content, and antioxidant activity of the extracts was assessed. Furthermore, anthocyanins were purified by adsorption–desorption and finally subjected to co-pigmentation with ferulic acid in order to increase color intensity and extend the product shelf life. The valorization of the juice pomace has the potential to increase profitability for the berry producers, and also minimize the environmental impact associated to waste management [33,34].

## 2. Materials and Methods

**Figure 1:**
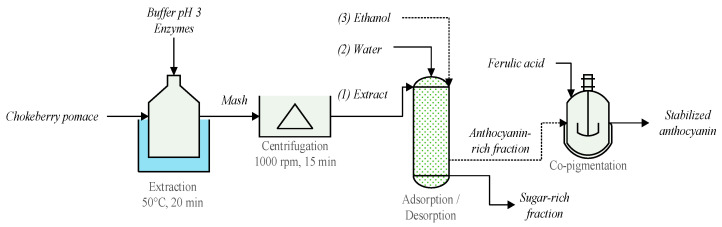
Proposed separation sequence for production of stabilized anthocyanins.

A schematic representation of the sequence of operations proposed in this study is shown in Figure 1.

### 2.1. Plant Material

Black chokeberry (*Aronia melanocarpa*) residual pomace from juice production was provided by Elkærholm (Egtved, Denmark). The freshly pressed pomace (moisture content 65 ± 1% wt.) was immediately frozen at −20 °C and thawed at 5 °C prior to experiments. All pomace batches were homogenized using a laboratory grinder GM 300 (Retch, Haan, Germany).

### 2.2. Enzyme-Assisted Extraction Process

The extraction experiments were carried out in a 1 L reactor equipped with a mechanical stirrer RZR 2050 (Heidolph, Schwabach, Germany, mixing velocity of 300 rpm) placed in an external water bath for temperature control. The enzyme formulations tested in the extraction experiments were Rohapect (AB Enzymes Gmbh, Darmstadt Germany), Fructozym BE, Fructozym Flash-C, and Fructozym Flux (Erbslöeh, Geissenheim, Germany). The extraction temperature, pH, enzyme dosage, and extraction time were selected based on the recommendations of the manufacturers. Shortly, 10 g of ground pomace were mixed with 500 g of pre-heated (50 °C) citrate–phosphate buffer solution (0.12 M, pH 3.0) containing the enzymes. The extraction proceeded for 20 min and aliquots of the mixture were collected, centrifuged (1000 rpm, 15 min), and stored at −20 °C for further analysis. All experiments were carried out in dim light.

### 2.3. Anthocyanin Purification via Adsorption–Desorption 

A non-ionic acrylic ester resin of moderate polarity (Amberlite^®^ XAD7HP, Sigma Aldrich, Søborg, Denmark) was selected. A total of 5 g of the resin was soaked in water, and loaded into a 10 mL glass column. Next, 10 *g* of centrifuged extract was loaded and demineralized water was run through until no sugars were detected in the eluent using a glucose detection kit (Precision Europe, Northampton, UK). Afterwards, the adsorbed anthocyanins were eluted by several fractions of ethanol 96% (*v/v*) acidified with 1% (*w/w*) of citric acid. The anthocyanin rich samples were pooled together, evaporated under vacuum at 35 °C using a rotary evaporator R-210 (Buchi, Flawil, Switzerland), and stored at −20 °C until further use.

### 2.4. Co-Pigmentation of Anthocyanins with Ferulic Acid

The water bath used for storage of the co-pigmented samples was previously described elsewhere [35]. Ferulic acid (Sigma Aldrich, Denmark) was selected as model co-pigment based on previous studies on co-pigmentation of cyanidins obtained from blueberry wine pomace and mulberry juice [36,37].

Anthocyanin dry extracts produced by adsorption–desorption were re-dissolved in acidified water (pH 3.0), and solid ferulic acid was added in order to reach four different anthocyanin: co-pigment molar ratios, namely 1:23, 1:48, 1:86, and 1:172. A sample without co-pigment addition was used as control.

Absorption spectra of the samples were recorded using a spectrophotometer DR 3900 (Hach, Düsseldorf, Germany) after 60 min, one week, two weeks, and one month in storage (5 °C, darkness).

The magnitude of the co-pigmentation effect was measured as the increase in absorption maximum wavelength (bathochromic shift, Equation (1)) and percentage increase of absorption intensity (hyperchromic shift, Equation (2)).

(1) $$\Delta \lambda_{max}=\lambda_{max}-\lambda_{max,0}$$

(2) $$\Delta A\;\left(\%\right)=\left[\frac{A_{max}-A_{max,0}}{A_{max,0}}\right]\cdot 100$$

where *λ_max_* and *λ_max_*_,0_ are the maximum absorption wavelengths (nm) of the co-pigmented and control samples at a given time, respectively; *A_max_* and *A_max_*_,0_ are the maximum absorbance intensities (mAu) of the co-pigmented and control samples at a given time, respectively.

### 2.5. Anthocyanin Identification and Quantification

Identification and quantification of the anthocyanins was carried out by high-performance liquid chromatography (HPLC) (HP 1200 series, Agilent Technologies Aps, Nærum, Denmark). Separation was performed on a reversed phase C18 column (Gemini 5 μ C18 110A, 250 × 4.6 mm i.d., Phenomenex Aps, Værløse, Denmark) operated at 25 °C, and equipped with a guard column (Security Guard System for C18, Phenomenex Aps, Værløse, Denmark). The eluents used were 0.05% v/v trifluoroacetic acid (TFA) in milliQ water (A) and 0.05% TFA in acetonitrile (B) in a gradient as follows: from 0–10% B in 1 min, from 10–20% B in 19 min, from 20–40% B in 20 min, from 40–80% B in 10 min, from 80–100% B in 2 min, and from 100%-0 B in 1 min. The eluent flowrate was 1 mL/min, and the sample injection volume was 20 µL. The detection was performed by a photodiode array detector measuring at 520 nm. Peak assignment was based on matching the retention times and spectral properties with external pure standards of cyanidin-3-galactoside, cyanidin-3-arabinoside, cyanidin3-glucoside (Extrasynthese, Genay, France), and cyanidin-3-xyloside (Carbosynth, Compton, CA, USA). All solvents and reagents were HPLC grade (VWR Prolabo, Søborg, Denmark). For absolute quantification, calibration curves based on known concentrations of the pure external standards were constructed (R2 > 0.990).

### 2.6. Total Phenolic Content Determination

Total phenolic content (TPC) of the samples was measured spectrophotometrically (DR 3900, Hach, Düsseldorf, Germany) according to the method described by Sun et al. [38]. Briefly, Folin–Ciocalteau reagent was diluted 10 times using deionized water, then 0.75 mL of diluted reagent was mixed with 0.1 mL of the sample and kept at room temperature for 5 min. Next, 0.75 mL of 2% sodium carbonate solution was added. The samples were kept for 15 min at room temperature, then the absorbance at 750 nm was measured. The TP concentration was derived from a standard curve of gallic acid and expressed as gallic acid equivalents (GAE) in mg GAE/L.

### 2.7. Antioxidant Activity Measurement

The antioxidant activity was determined by the 2,2-diphenyl-1-picrylhydrazyl radical (DPPH) assay, as described by Scherer and Godoy [39]. A total of 3.9 mL of 0.124 mM DPPH methanolic solution was added to 0.1 mL of sample. A control sample was prepared by mixing 3.9 mL of the DPPH solution with 0.1 mL of pure methanol. All samples were incubated for 7 h at room temperature and dim light. The absorbance at 516 nm was then measured, and the inhibition percentage (IP) of the DPPH· radical was calculated using Equation (3).

(3) $$IP\;\left(\%\right)=\left[\frac{A_{516,0}-A_{516}}{A_{516,0}}\right]\cdot 100$$

*A*_516,0_ is the absorbance at 516 nm of the control sample at time zero, and *A*_516_ is the absorbance at 516 nm of the sample after 7 h incubation.

### 2.8. Statistical Analysis

The extraction experiments were carried out in triplicates, and the co-pigmentation experiments in duplicates. The values are expressed as the mean ± standard deviation. Statistical significance (*p* < 0.05) was assessed by one-way analysis of variance (ANOVA) and Tukey’s test using IBM SPSS Statistics version 26 (IBM Corp., Armonk, NY, USA).

## 3. Results

### 3.1. Anthocyanin Profile in Chokeberry Pomace Extracts

Four monoglycosylated anthocyanins were identified in all chokeberry pomace extracts in the following proportions: cyanidin-3-O-galactoside (62%), cyanidin-3-O-glucoside (4%), cyanidin-3-O-arabinoside (30%), and cyanidin-3-O-xyloside (5%). The proportion of the individual anthocyanins in the extracts was not affected by the enzymatic treatment in any of the cases.

### 3.2. Enzyme-Assisted Extraction

The extractions were performed for 20 min, since extended treatment resulted in anthocyanin loss. The results obtained for the extraction experiments are shown in Table 1. Liquefaction of the pomace by enzymatic treatment was confirmed by the increased soluble matter released to the solution, as given by the increase in Brix degrees from 1.1 to 2.2. The absolute anthocyanin content increased in all enzyme-treated samples. However, the results showed a large variation among experiments, which was probably due to the heterogeneity of the feedstock. For this reason, the absolute increase cannot be considered significant at a confidence level of 95%. Furthermore, none of the enzyme-assisted extractions showed an increase in antioxidant capacity when compared to the control sample (*p* < 0.05). The enzyme formulation Rohapect resulted in an increase of the total phenolic content of the extract, while Fructozym Flux was the only one that resulted in a lower total phenolic content.

Several studies have reported an increase of phenolic extraction yields through enzymatic treatment of berry by-products [40,41]. However, in the case of anthocyanin extraction, the results seem to be very much dependent on the type of plant matrix used. For grape wine pomace, Maier et al. [42] observed an increase in anthocyanin extraction yield using Novoferm 106 and Cellubrix L. Similar results were observed for grape wine pomace using Vinozym EC [43]. However, in the case of blueberry pomace, enzymatic treatment with pectinases seems to have little effect on the anthocyanin extraction yield. Landbo and Meyer [41] did not observe an effect on anthocyanin extractability when using Pectinex BE and Novozym 89, and the effect was detrimental when using Macer8 FJ and Macer8 R. Lee and Wrolstad [44] reported no effect on anthocyanin extraction when using nine different commercial juice processing enzyme formulations. In a study on chokeberry pomace, Kitrytė et al. [40] observed an increase in soluble matter, total phenolic content, and antioxidant activity after enzymatic treatment of the pomace using Viscozyme L and CeluStar XL.

A possible explanation for this variability is that anthocyanins in berries are mostly found entrapped in the cell vacuoles, and not embedded in the cell wall [45]. Enzymatic degradation of the cell wall components plays an important role on cell disintegration and release of vacuolar and cell wall bound phenolics. However, for some plant matrices, an appropriate mechanical size-reducing strategy could in principle be able to achieve the maximum extractability for a given set of extraction conditions (solvent, temperature, pH, ionic strength, time). Once the solid and liquid phases are in equilibrium, further cell disintegration does not increase anthocyanin extractability since there is no driving force for the mass transfer process. Furthermore, once released from the vacuoles, the anthocyanins come in contact with the plant cell wall components and endogenous enzymes present in the suspension. Released anthocyanins have been reported to bind cellulose and cellulose-pectin composites via ionic and hydrophobic interactions, resulting in anthocyanin depletion from the solution [46]. This effect could, in principle, mask the increase in anthocyanin extractability (if any) due to the increased polysaccharide binding surface available. This observation emphasizes the importance of utilizing suitable purification processes that minimize the contact time between extracted anthocyanins and the plant cell wall residues present in the extraction media.

### 3.3. Purification of Anthocyanins by Adsorption–Desorption 

**Figure 2:**
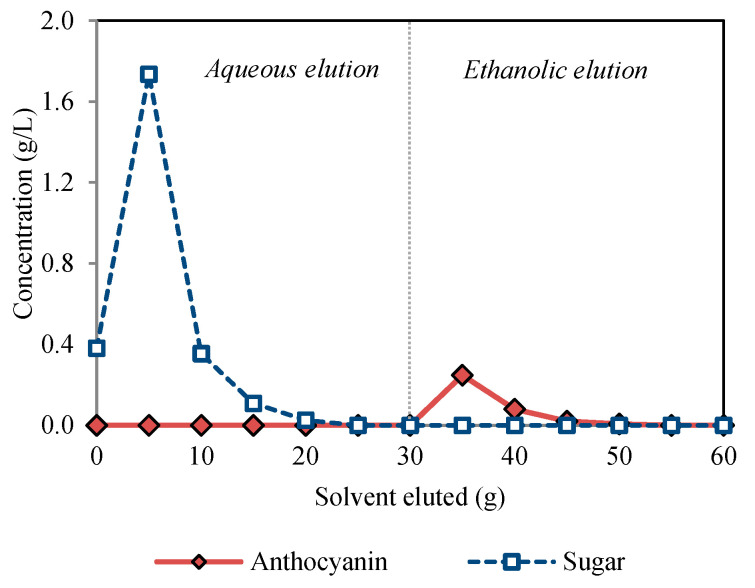
Elution profiles of sugars and anthocyanins after demineralized water (0–30 g) and acidified ethanol (30–60 g) elution of chokeberry extracts absorbed over Amberlite^®^ XAD7HP resin.

The elution profiles for both aqueous and ethanolic eluents in the purification experiment are shown in Figure 2. The resin Amberlite XAD7HP^®^ showed favorable adsorption and desorption performances for anthocyanins, which are attributed to their similar polarity and its high solid phase surface area. These results agree with other studies using the same type of resin [7,47]. The sugars in the extract were washed out in the aqueous eluent (deionized water), where no anthocyanins were detected, showing that the interaction between the anthocyanin and the resin is capable of withstanding the water elution. Ethanolic elution, on the other hand, provided efficient anthocyanin desorption with a recovery of nearly 96%.

### 3.4. Co-Pigmentation of Anthocyanins with Ferulic Acid

Endogenous or added sugars have a protective effect on anthocyanin stability due to reduced water activity in the solution [48,49]. During purification of anthocyanins via adsorption, the protective effect of sugars is lost, and suitable stabilization processes such as co-pigmentation become of utmost relevance to preserve anthocyanin content and color.

**Figure 3:**
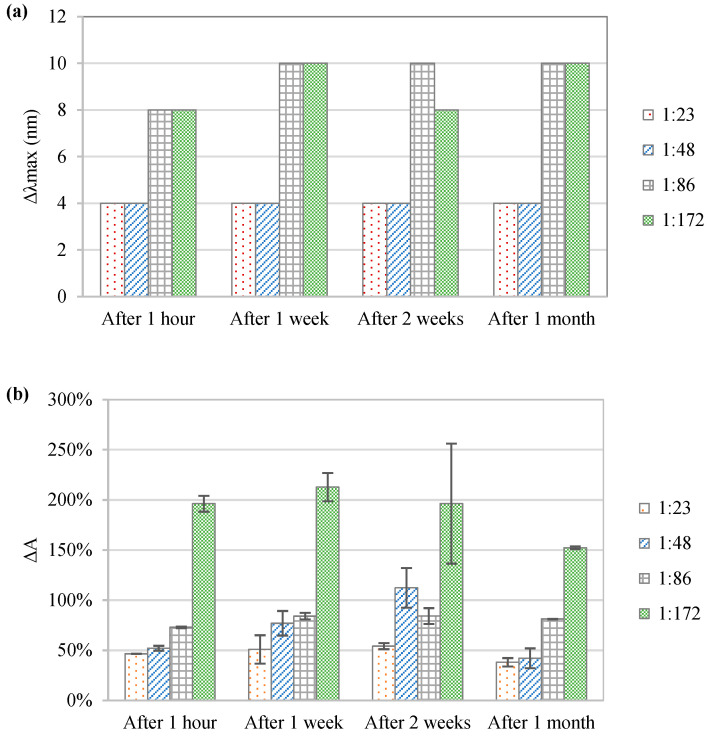
Effect of the anthocyanin: co-pigment ratio on the preservation of chokeberry extracts during 1 month storage at 5 °C: (**a**) absorption maximum shift (Δ*λmax*) and (**b**) increase of absorbance intensity (Δ*A*%).

As shown in Figure 3, the co-pigmentation effect increased with the anthocyanin: co-pigment molar ratio. The control samples had an absorption maximum (*λ_max_*_,0_) of 512 nm. The strongest immediate bathochromic shift was observed at the anthocyanin: co-pigment molar ratio of 1:86 and 1:172, where Δ*λ_max_* was in both cases ~10 nm. The occurrence of the bathochromic shift confirms that co-pigmentation does indeed take place, by showing that the spectral properties of the pigment are affected by the formation of the complex.

The hyperchromic effect increased drastically with the amount of co-pigment added. Our study shows that even after a month of storage, all co-pigmented samples showed a higher absorbance intensity than the respective control samples. The largest hyperchromic shift (200% increase) was observed for the highest anthocyanin: co-pigment molar ratio tested (1:172). This ratio is, however, not realistic for a practical food application scenario due to the high quantity of co-pigment required and its cost. Based on similar observations, Klisurova et al. [50] recommended the use of herbal extracts such as lavender or green tea, which contain the phenolic co-pigments, instead of using the pure compounds. The optimal anthocyanin: co-pigment ratio results as a compromise between co-pigment cost and the pigment stability gain obtained in terms of color enhancement and extended shelf life [51]. Further work on the screening of co-pigments and concentrated herbal extracts to be used as co-pigment sources is recommended.

## 4. Conclusions

The present case study describes a process for the recovery of anthocyanins and other phenolics from chokeberry juice pomace that combines extraction, purification, and stabilization. The enzymatic treatment increased the anthocyanin concentration in the treated extracts, even though high variations in the anthocyanin content were observed. Purification of the anthocyanins was achieved via adsorption–desorption using an acrylic resin, which resulted in a 96% recovery of the extracted anthocyanin. Stabilization was achieved by co-pigmentation with ferulic acid, which enhanced color intensity and stability during storage of the solutions for up to one month. This case study aims at providing a possible valorization route for chokeberry pomace with an overall sequence from raw material to final product, and thus represents one more step towards real implementations of processes for waste valorization. Further investigation in the details of each unit operation and process alternatives is necessary to obtain a wide overview of the process possibilities, and most importantly to compare them in terms of profitability and sustainability.

## Figures and Tables

**Table 1:** Total anthocyanin content (TAC), total phenol content (TPC), inhibition percentage of the DPPH radical (IP), and Brix degrees for the chokeberry extracts.

Enzyme Formulation	Enzyme Dosage (mL/ton)	TAC (mg/L)	TPC (mg GAE/L)	IP (%)	Brix
**Control**	0	233.5 ± 24.7 ^a^	517.1 ± 6.5 ^a^	48.0 ± 0.7 ^a^	1.1 ± 0.1 ^a^
**Rohapect**	500	264.2 ± 17.3 ^a^	573.3 ± 14.9 ^b^	47.5 ± 1.6 ^a^	2.1 ± 0.1 ^b^
**Fructozym BE**	400	283.8 ± 20.0 ^a^	505.6 ± 10.5 ^a^	49.9 ± 1.5 ^a^	2.2 ± 0.0 ^b^
**Fructozym Flash-C**	400	308.3 ± 10.4 ^a^	492.6 ± 9.3 ^ac^	43.6 ± 0.5 ^a^	2.2 ± 0.0 ^b^
**Fructozym Flux**	400	332.4 ± 60.6 ^a^	469.1 ± 2.2 ^c^	44.6 ± 1.6 ^a^	2.2 ± 0.1 ^b^

The values are mean ± standard deviation based on three independent replicates (n = 3) of each experiment. Different superscript letters within the same column indicate significant differences (*p* < 0.05).

## Data Availability

The data are available from the corresponding author.
